# Provider Perspectives on Sleep Apnea from Appalachia: A Mixed Methods Study

**DOI:** 10.3390/jcm11154449

**Published:** 2022-07-30

**Authors:** Robert Stansbury, Toni Rudisill, Rachel Salyer, Brenna Kirk, Caterina De Fazio, Adam Baus, Shubekchha Aryal, Patrick J. Strollo, Sunil Sharma, Judith Feinberg

**Affiliations:** 1 Section of Pulmonary, Critical Care, and Sleep Medicine, West Virginia University, Morgantown, WV 26506, USA; shubekchha.aryal@hsc.wvu.edu (S.A.); sunil.sharma@hsc.wvu.edu (S.S.); 2 Department of Medicine, University of Pittsburgh, Pittsburgh, PA 15213, USA; strollopj@upmc.edu; 3 School of Public Health, West Virginia University, Morgantown, WV 26501, USA; trudisill@hsc.wvu.edu (T.R.); bok0001@hsc.wvu.edu (B.K.); caterina.defazio@hsc.wvu.edu (C.D.F.); abaus@hsc.wvu.edu (A.B.); 4 Department of Medicine, West Virginia University, Morgantown, WV 26506, USA; rachel.salyer@hsc.wvu.edu (R.S.); judith.feinberg@hsc.wvu.edu (J.F.); 5 Department of Medicine, Veterans Affairs Pittsburgh, Pittsburgh, PA 15240, USA

**Keywords:** respiratory health disparity, rural health, obstructive sleep apnea, mixed methods, community engaged research

## Abstract

West Virginia (WV) has the highest rates of obesity and cardiopulmonary disease in the United States (U.S.). Recent work has identified a significant care gap in WV for obstructive sleep apnea (OSA). This OSA care gap likely has significant health implications for the region given the high rates of obesity and cardiopulmonary disease. The purpose of this mix methods study was to identify barriers that contribute to the rural OSA care disparity previously identified in WV. **Methods:** This study used mixed methods to evaluate the barriers and facilitators to management of OSA at Federally Qualified Health Centers serving communities in southern WV. Focus groups were conducted at federally qualified health centers with providers serving Appalachian communities. Participants also completed the validated Obstructive Sleep Apnea Knowledge and Attitudes (OSAKA) questionnaire to gain insight into provider knowledge and beliefs regarding OSA. EMR analysis using diagnostic codes was completed at the sites to assess OSA prevalence rates. The same individual served as the interviewer in all focus group sessions to minimize interviewer variability/bias. Our team checked to ensure that the professional transcriptions were correct and matched the audio via spot checks. **Results:** Themes identified from the focus groups fell into three broad categories: (1) barriers to OSA care delivery, (2) facilitators to OSA care delivery, and (3) community-based care needs to optimize management of OSA in the targeted rural areas. Questionnaire data demonstrated rural providers feel OSA is an important condition to identify but lack confidence to identify and treat OSA. Evaluation of the electronic medical record demonstrates an even larger OSA care gap in these rural communities than previously described. **Conclusion:** This study found a lack of provider confidence in the ability to diagnose and treat OSA effectively and identified specific themes that limit OSA care in the communities studied. Training directed toward the identified knowledge gaps and on new technologies would likely give rural primary care providers the confidence to take a more active role in OSA diagnosis and management. An integrated model of care that incorporates primary care providers, specialists and effective use of modern technologies will be essential to address the identified OSA care disparities in rural WV and similar communities across the U.S. Community engaged research such as the current study will be essential to the creation of feasible, practical, relevant and culturally competent care pathways for providers serving rural communities with OSA and other respiratory disease to achieve health equity.

## 1. Introduction

Appalachia—especially West Virginia (WV)—persistently has the highest rates of obesity, cardiovascular disease, and smoking-related pulmonary illness in the United States [1,2,3]. Despite federal support and other programs aimed at improving care in rural communities, these health disparities persist [4]. Among those with cardiopulmonary disease, research suggests significant improvements in health outcomes with treatment of obstructive sleep apnea (OSA) [5,6,7,8,9]. Given these highly prevalent comorbid conditions, identification and treatment of OSA is of particular importance in improving health outcomes in disadvantaged rural communities. 

OSA prevalence in adults is increasing and recent work demonstrates nearly a billion people worldwide have undiagnosed sleep apnea suggesting a prevalence of 13% [10,11]. However, there is significant geographical variation in OSA prevalence, with some countries having a prevalence rate that exceeds 50% in the adult population [11]. This rise in OSA prevalence has been attributed in part to the increased prevalence of obesity, a key risk factor for OSA, and no region in the U.S. has been more affected by the obesity epidemic than Appalachia [12]. According to the CDC’s Behavioral Risk Factor Surveillance System (BRFSS), WV has the highest obesity prevalence rate at almost 40% [1]. Previous work suggests that OSA is under-recognized and ineffectively managed in rural WV communities [13,14]. A large study analyzed the WV Medicaid database covering approximately 1/3 of the state’s adult population and found that only 8% carried a diagnosis code for OSA compared to an expected prevalence in this population of 25% [15]. This OSA care gap in WV is likely related to healthcare disparities in rural Appalachian communities that lack access to specialty care.

Like many rural areas, WV has a dearth of specialty providers, and management of chronic respiratory conditions such as OSA typically falls to primary care providers. For this study, we conducted: (1) a community-engaged research project to assess the knowledge and beliefs of primary care providers with regard to OSA and how these may impact their decision to screen and treat OSA, (2) quantitative analysis of diagnostic patterns at the targeted Federally Qualified Health Centers (FQHCs). These community-based health care providers receive funds from the Health Resources and Services Administration to provide primary care services in underserved areas [16]. We hypothesized that unidentified barriers to care for OSA contribute to the rural OSA care disparity previously identified in WV.

## 2. Materials and Methods

This study used mixed methods to evaluate the barriers and facilitators to provider management of OSA at FQHCs serving communities in southern WV, an area with some of the poorest healthcare outcomes and healthcare disparities in the U.S. [4]. West Virginia has a population of ~1.7 million inhabitants [17]. Ninety-three percent of the population is Caucasian [17]. Unfortunately, 15.8% live in poverty and 8.3% do not have health insurance. Approximately 1/3 of the state’s population has Medicaid insurance coverage [15]. On a per capita basis, there are about 69 PCPs per 100,000 persons in West Virginia. The specific FQHCs in this study were targeted for multiple reasons, including: (a) providing care for a rural population with significant healthcare disparities, (b) having providers who expressed interest in the study during preliminary discussions with our team, and (c) having support from the FQHC’s executive leadership to pursue clinical research to improve care for the community served. Data sources included qualitative data from focus groups, questionnaires, and analysis of the FQHCs’ electronic medical record. This study was reviewed by the West Virginia University IRB (Exempt Protocol 2009107631) and supported by funding through the West Virginia Clinical and Translational Sciences Institute (NIH/NIGMS 5U54GM104942-03).

### 2.1. Qualitative Methods

Targeted providers included primary care physicians and advanced practice providers who were recruited for participation through the support of the West Virginia Practice Based Research Network (WVPBRN). The WVPBRN is a group of primary care clinicians and practices partnered with research centers (such as West Virginia University, the WV School of Osteopathic Medicine, and Charleston Area Medical Center) who work together to answer community-based healthcare questions and translate research findings into meaningful everyday practice. The WVPBRN contacted executive leadership and the chief medical officer at each FQHC, who subsequently offered participation to all providers. Provider focus groups were scheduled with the support of the WVPBRN and local collaborators championing our efforts at each of two clinical sites at two FQHCs, for a total of four sites.

All providers at the targeted clinic sites were included in the study (except one provider who was on vacation at the time of the focus group). No subspecialists participated in these focus groups; there were no exclusion criteria. Participants received USD 100 for their time and effort.

Each focus group had 3-to-5 participants (*n* = 14). A semi-structured interview guide was developed to assess understanding of provider knowledge and comfort level with OSA management as well as to explore their perspectives on the barriers and facilitators to OSA care in their rural communities (Table 1). Questions were developed based on input from rural practitioners and previous research [13,14,15]. Focus group sessions lasted approximately 45 min, were audio recorded and professionally transcribed. Professional transcription was completed with NVivo (QRS International Data^C^, Ruggell, Liechtenstein). Saturation was achieved by the fourth focus group, where no new themes emerged. The same individual served as the interviewer in all focus group sessions to minimize interviewer variability/bias. Our team checked to ensure that the professional transcriptions were correct and matched the audio via spot checks. 

### 2.2. Quantitative Methods

Three data sources were utilized: demographic questionnaires, the validated Obstructive Sleep Apnea Knowledge and Attitudes (OSAKA) questionnaire [18], and the site electronic medical record (EMR). All focus group participants completed demographic questionnaires that captured gender, age, race, professional certification, board certification (if applicable), years in practice, and years in practice at the current FQHC. The OSAKA questionnaire was used to determine providers’ OSA knowledge, using 18 true-false statements with a third option of “don’t know.” This portion is scored for correctness, with a higher score indicating greater OSA knowledge. Then, using a five-point Likert scale, providers rate their level of agreement with five statements. The first two statements are about the importance of OSA (1 = not important, 5 = extremely important) and next three statements are about their confidence in identifying and managing OSA (1 = strongly disagree, 5 = strongly agree). Higher scores for these two portions indicate greater rating of the importance of OSA and provider confidence in diagnosing and managing OSA. Questionnaires were completed in paper format. The data were then de-identified and stored in a HIPAA-compliant, secure Research Electronic Data Capture (REDCap) database (hosted at WVCTSI). Finally, we assessed OSA diagnosis rates at one of the targeted FQHCs through EMR interrogation for the period between 1 July 2019 and 19 October 2021. We queried adult patients with an ICD-10 code for sleep apnea during the reporting period [ICD-10 codes G4731 Primary Central Sleep Apnea; G4733 Obstructive Sleep Apnea (adult) (pediatric); G4739 Other Sleep Apnea].

### 2.3. Statistical Analysis

#### 2.3.1. Qualitative Analysis: Focus Groups

Focus group transcripts were analyzed using content analysis to identify themes relating to facilitators and barriers of sleep apnea management. Two coders thoroughly read and reread the transcripts, and each coder independently developed code words/phrases to label thoughts or concepts that emerged. The research team met with the coders to compare their initial coding schemes and reached consensus on how to code thoughts and concepts. These codes were then operationally defined and documented in a data dictionary within the NVivo software. After all transcripts were coded, our team sorted and collapsed the operationally defined codes into broader, more encompassing themes or subthemes. Inter-rater reliability was evaluated using Cohen’s kappa statistic. It was determined a priori that if a minimum kappa of 0.8 was not met, the coding process would occur iteratively until that value was reached. After core themes were determined, the transcripts were reread to ensure that these themes accurately depicted the data. Data management, including searching, coding and categorization of the text from transcripts, was completed with NVivo (V.12).

#### 2.3.2. Quantitative Analysis

Questionnaires: Demographic data were analyzed descriptively as frequencies and percentages. Continuously scaled measures were summarized by means, standard deviations, medians, and ranges. The OSAKA questionnaire was used to determine providers’ OSA knowledge, importance, and confidence. Provider responses to OSA knowledge items were analyzed for correctness. This dataset was analyzed with SPSS Windows version 28 (SPSS Inc., Chicago, IL, USA). 

Electronic Medical Record: This analysis was completed in collaboration with West Virginia University Office of Health Affairs who have an agreement with the FQHCs to use the EMR data source for approved research projects. This partnership provides WVU faculty access to anonymized data. Record-level claims data from 2021 were aggregated at the individual level to assess the overall prevalence of sleep apnea among adult patients at the FQHC. We calculated the prevalence of OSA diagnoses by dividing the number of patients with a sleep apnea ICD-10 code by the number of unique patient encounters during the study timeframe. Descriptive statistics provided details on age, sex, race, and ethnicity for patients with an OSA diagnosis. JMP Statistical Discovery Software version 15 was used for this analysis (SAS Inc, Cary, NC, USA). 

## 3. Results

### 3.1. Questionnaire Results 

A total of 14 providers participated in the focus group sessions. Participants’ demographic characteristics are shown in Table 2. Eight participants were advanced practice providers and six were physicians

They had 16.2 mean years in practice overall and 8.7 mean years in practice at the FQHC; however, there was a wide range of years in practice. The majority of participants were female. All physicians were board- certified in Family Medicine.

Regarding the OSAKA questionnaire, the mean OSA importance rating was 9.1 for all providers with a highest possible score of 10 from the two Likert questions on the OSAKA regarding disease importance. The average OSA confidence rating was 9.6 with a highest possible score of 15 from the three Likert questions on the OSAKA regarding disease confidence in disease management. Knowledge scores ranged from 11 to 16 correct answers out of 18. The average number of correct answers for all providers was 14 (SD 1.6). Table 3 shows correct participant responses to each individual item. No provider answered all the knowledge questions correctly. The most common items answered incorrectly were item 3- OSA epidemiology (28.6% respondents answered correctly), Item 4- OSA symptoms (57.1% respondents answered correctly), and item 8- OSA treatment (28.6% respondents answered correct). 

### 3.2. Focus Group Themes

Themes identified from the focus groups fell into 3 broad categories (Table 4): (1) barriers to OSA care delivery, (2) facilitators to OSA care delivery, and (3) community-based care needs to optimize management of OSA in the targeted rural areas.

#### 3.2.1. Theme 1: Barriers to OSA Care Delivery

**OSA Care Access**: A major concern for providers was whether their patients could access care for OSA. Providers reported making referrals for OSA testing or specialist evaluation, but patients often did not follow-up. Reasons for lack of follow-up include lack of transportation or cost of transportation, scheduling difficulties, not wanting to stay overnight for a sleep study, patients not “trusting” other providers and only wanting to be treated by their primary care provider, and important aspects of the social determinants of health for the community including poverty, low health literacy and low educational levels. Even when patients followed through with referrals, it was difficult for the providers and patients to get feedback from the appointments and/or test results. Providers described the issues with patients’ access to care:

“And lack of transportation. Most of the patients I see, they want to go close to here and there’s only one facility here [sleep specialist] and sometimes scheduling is a nightmare just because you cannot get ahold of anybody there.”

“[T]here’s nothing here locally; they have to go Charleston or a lot of the other what have you and a lot of these people do not have cars or can’t pay four zillion dollars, if they can’t afford for somebody to transport them, then that’s the problem.”

“The constraints that I’ve seen though, nobody wants to do an overnight sleep study and some of the insurances don’t want to pay for the home sleep studies, they only want them to go to a site and nobody wants to do that…”

Treatment for OSA is also described as a challenge. Many patients will not accept continuous positive airway pressure therapy (CPAP). Those who do undergo a trial of CPAP therapy may encounter problems, but there is not good local support from durable medical equipment (DME) companies to address these issues. Patients don’t communicate if they are having problems, and then just stop using CPAP. As providers explained:

“I don’t know if it’s related to the [DME] company versus a patient, but they [the patient] will usually come to you first and say, ‘Oh, I couldn’t get that last thing to work or whatever’. So, I don’t know if it’s because on that end they’re [the DME company] hard to get in contact with or they [the patient] dropped the ball.

“[T]here’s certainly some patients who don’t do well with CPAP and they probably don’t tell anybody and just don’t get treatment.”

“They [patients] don’t tell you if it’s actually not working I think.”

“The patients are not very successful with trying to contact the companies once they’ve dropped the equipment…. It’ll be stuck on us to try to get back in contact with the third [party] company.”

**Variable Provider Knowledge/Beliefs About OSA**: In general, providers reported that screening patients for OSA was a low priority, but did note that there seems to be increasing awareness in the local medical community. Symptoms that may trigger OSA screening included fatigue/sleepiness, choking/coughing at night, spouse complaints of snoring, and chronic headaches. Hypertension was the mostly commonly reported disease that prompted OSA screening, although there was significant variability in reported conditions that led to OSA evaluation. Most providers will not evaluate patients with these conditions unless the patient has sleep-related complaints. As providers explained:

“Everybody in my practice complains of fatigue. And almost everybody complains of fatigue and fatigue is such a broad diagnosis. A lot of them you’ll ask them, ‘Do you snore’? ‘Well, I don’t know if I do or not’. They’ll giggle and say, ‘I don’t listen to myself sleep’”.

“I have to say in all honesty I think over my career I’ve probably done a lousy job of screening for sleep apnea. And I’ve probably only referred people for testing when they said they were sleepy. And, um, and even then, I think it was low down on my list of things to consider to do a sleep study and to think about sleep apnea.”

“I don’t think I’m screening and I don’t think I’m doing follow up, um, uh, gosh, questions related to it. It’s really not on, on my radar.”

“I, uh, learned a little bit more about sleep apnea in the past several years from continuing education class. But I think there’s a lot I don’t know.”

“If you mean do I have a questionnaire that I ask my patients or have them complete, the answer is no. But if you ask me if I screen the patient, I do. If you’re obese and hypertensive, if you’re obese and complain of not being able to sleep well at night, or that you wake up coughing or choking along with GERD symptoms, I mean, I screen that way.”

Providers did not feel comfortable reviewing and interpreting sleep study data and “don’t trust” the results of a negative home sleep test. Providers did not feel comfortable managing CPAP machines including trouble-shooting common issues and reviewing data downloaded from the device. Providers will order a portable sleep test, but will leave treatment decisions up to a specialist. Some providers were willing to refill prescriptions for CPAP supplies if a patient is doing well, but were not comfortable making any adjustments to the CPAP device. In general, there was limited knowledge about CPAP alternatives. As one provider explained:

“I was going to say I can read it [the results] and understand some. The last one I had, I thought it was kind of difficult to interpret the gist of the wording and stuff on the report.”

“No, I feel more comfortable when I get the results and seeing its positive to send them [patients] somebody that can do that, not that I wouldn’t, if I had the training, feel like I could do it. I’ve just never had the training.”

“I just don’t know like, you know, change in settings, you know, how many… if they’re [patients] still having episodes of apnea, you know, all of the adjustments you have to make on the machines, I really don’t even know where to start.”

“I just refer and let them [specialists] manage it. I mean, if uh… Even in the dental appliances, I would not know where to… who to approach to about this fitting mouthpieces and things that they do, so I just, I just assume the uh, sleep center knows all that.”

“I only ask them [patients] the last time that their equipment was replaced, like their face mask and that type of stuff, but outside of that I would have no clue.”

**Cost of OSA Care:** High cost and variable insurance coverage are other sub-themes impacting OSA care in the targeted rural communities. Some insurances will not cover specialty referral and require a home sleep test through the primary care provider leaving the provider with results they do not understand. Some specialty providers will not accept patient insurance due to low reimbursement. Testing and equipment for OSA treatment are often denied by insurance. Insurance requirement for yearly OSA follow-up was also a noted barrier for many patients who cannot afford travel to specialty clinics or do not have access to transportation. As providers discussed:

“14% of people are uninsured and then you heard people comment that some of the insurances don’t cover this specific test, or that specific, so definitely healthcare coverage is also a barrier.”

“People’s ability to get a test. And, and sometimes, um, they can’t afford it. I, I do, um, patient physicals and the, the DOT [Department of Transportation] requires that everybody who uses a CPAP get a, an annual sleep study. And, um, we really run into a problem that a lot of these guys can’t, or ladies, can’t afford the follow-up sleep study or the follow-up visit. I’m not sure why it’s not well covered by insurance.”

“[T]he other thing is I noticed even before I started doing those physicals that some insurers prefer to just do the overnight sleep study without, uh, referral to a specialist, and so then I was left holding the results and was not real happy about that. So, so to me, the barrier, the biggest barrier is cost and coverage.”

“And then they [dental professional] want some ridiculous amount for the, the [oral] appliance. People don’t have the money.”

“And, and, and besides those that pay for it, it’s a question of does the specialist accept that kind of insurance?…. Because if the reimbursement is low, and I think that’s an issue with our person in XXX.”

#### 3.2.2. Theme 2: Facilitators to OSA Care Delivery

Providers were able to identify certain characteristics that improved the likelihood of a patient successfully navigating the local OSA care pathway leading to diagnosis and effective treatment.

**Specialty Referral Access**: Rural providers with good access to specialty care clinics and DMEs felt more comfortable with OSA management although they still referred patients to specialists. In general, these providers were comfortable ordering sleep tests and CPAP supplies and would refer patients to DMEs for CPAP trouble-shooting. Proximity to specialty centers mitigated transportation issues for some patients. Increased access to and Medicaid coverage of home sleep testing also facilitated OSA care delivery. One provider explained the relationship with a local specialist and their DME company:

“They [specialist and DME] pretty much take care of all that stuff. As long as they do all that, then I’m pretty comfortable with that. But if they don’t, which I’ve never seen them not, we titrate the machines and do all that. And then they will follow them only if they have an underlying COPD or something like that, I think pulmonary gets involved. But other than that, once they’re diagnosed and their machines are titrated and all that good stuff, they [patients] just stay with us.”

**Patient Characteristics:** Providers felt that patients are generally receptive to discussion of OSA and open to referral for evaluation. Patients have the most trust in their local provider and when their provider takes time to discuss OSA testing and CPAP treatment they are more likely to follow-up with the referral. Providers also stated that there is more awareness in the community with regard to OSA which facilitates diagnosis and successful management. As one provider explained the receptivity of patients to treatment for OSA:

“I’ve never had any patient that closed the door on that discussion with me in my practice. They’re usually very open. They feel so bad, and they want to figure out why, whether they believe that or not is the question, but they’re open to the possibilities [of getting assessed for OSA].”

Theme 3: Community-based Care Needs to Improve OSA Management in the Targeted Rural Areas.

**Community Programming:** Providers felt that a special community program for OSA would need to focus on patient education. Patients are not aware of the adverse impact of OSA and poor sleep on health. Patients tend to just accept bad sleep as something that is not changeable. In general patients are hesitant to share what’s wrong or that there is a problem, especially if they are male as Appalachian culture prizes toughness as a feature of masculinity. As one provider explained the receptivity of patients to treatment for OSA:

“A lot of them [patients] are these big burly gentlemen that are very manly, and they don’t want to say that, ‘Yeah. Well, maybe something’s not right. I’m not tired’. But yet they can’t lift five pounds, because they are so tired. So, you have this, no disrespect, but this ‘man mentality’ to some of these guys.”

**Provider Preferences**: Providers felt they could develop comfort with home sleep testing but would need specific education about CPAP devices. In general, providers would not mind oversight of home tests, although some felt that the specialist should order the appropriate diagnostic tests. Providers noted that patients will often call them with CPAP issues and felt comfortable addressing easy equipment issues, but many times felt that they needed more training to respond.

“I mean, it’s my responsibility, but since you’re conducting this study, I would be interested in more education on it [OSA and CPAP]”.

**Educational Needs:** Providers wanted more education regarding OSA screening, diagnosis and management. The main concern was the limited time for patient visits and whether OSA management could be practically accomplished in a rural primary care clinic. As one provider explained:

“I probably would consider learning how to manage it [OSA] more myself and prescribe CPAP. Um, I’m sort of hesitant to say that ‘cause I, you know, you, you worry about getting overwhelmed with things. My worry with that would be the clinical time to do it”.

### 3.3. EMR Analysis

The EMR analysis identified 21,701 unique patients who sought care from July 1 2019 to October 19 2021. Among those patients, 507 (2.3%) had an ICD-10 code (G47.31, G47.33, G47.39) for sleep apnea. Almost all (99.6%) were diagnosed with “obstructive sleep apnea (adult) (pediatric)” as represented by ICD-10 code G47.33. We compared this result to expected prevalence rates based on national datasets and previous work assessing OSA prevalence in the WV Medicaid population (Table 5). The expected prevalence for OSA in the targeted population is 25% [15]. Our analysis demonstrated a prevalence rate of 2.3% based on diagnostic coding.

## 4. Discussion 

**Figure 1:**
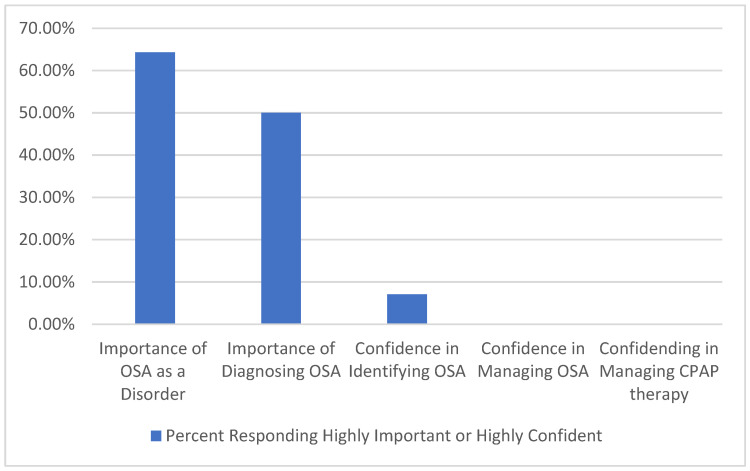
Percentage of Participants (*n* = 14) Responding OSA is Highly Important or Have High Confidence in OSA Management.

Our results showed that primary care providers from Appalachia feel OSA is an important disease to consider and have reasonable knowledge but lack the confidence to assume primary management (Figure 1). The OSAKA questionnaire also identified some specific knowledge gaps that could be easily addressed through targeted training. Utilizing a low-cost education and training intervention identified by the focus group sessions may have a significant impact on increasing the OSA workforce. While providers reported not being comfortable reviewing sleep study results or managing positive airway pressure therapy, studies have demonstrated that primary care providers can be effectively trained in the management of OSA including the use of these technologies with non-inferior outcomes compared to specialists’ care [19,20,21]. A challenge in the clinics we evaluated is the high prevalence of cardiopulmonary disease in the population. These patients were excluded from these previous studies assessing primary care management of OSA. The current American Academy of Sleep Medicine guidelines recommend management of OSA in patients with significant cardiorespiratory disease be through a specialized sleep center [22]. However, the COVID pandemic has led to paradigm shift in this thinking and these complex patients may be managed by primary care providers in collaboration with specialists through telemedicine [23].

Our findings suggest that the current OSA care model in rural WV has significant barriers that prevent successful navigation through the diagnostic testing and treatment pathways. The majority of providers stated they refer to specialty sleep centers or specialists for diagnostic testing and treatment, but given healthcare access issues in these communities related to patient financial constraints, transportation issues and geographic isolation, this approach alone cannot address the OSA disparities in rural WV and in other rural settings. Similar challenges have been identified in rural communities both inside and outside the United States [14,24,25,26]. Implementation of a three-pronged strategy may provide a possible solution to this issue of limited access decreasing this inequity in the targeted region and other rural areas. This strategy includes: (1) training of local providers in OSA care (2) distribution of portable sleep testing through rural health clinics, and (3) management of auto-titrating continuous positive airway pressure (APAP) treatment program through rural health clinics in partnership with local DMEs. Implementation of this three-pronged approach would likely require some support from specialists. Fortunately sleep medicine has been at the forefront of telemedicine for years including remote diagnostics, teleconsultation and telemonitoring of therapy [27,28,29]. These well-developed technologies could not only address rural care disparity for sleep apnea, but could also serve as a model for addressing respiratory health inequity in other disease states and communities [30]. 

The providers in the current study felt targeted education on OSA management including training on portable sleep testing and APAP management could empower them to take primary ownership of OSA care and they would be enthusiastic for this training. The main concern with OSA management was with regard to feasibility; specifically lack of healthcare provider time and clinic resources. This important theme warrants further consideration. Based on recent data OSA is becoming as prevalent as other common conditions such as DM and HTN managed through primary care [11]. A mindset change for primary care and paradigm shift in OSA management pathways may be required. As with DM and HTN, PCPs will need to manage “straightforward” OSA cases to address this growing epidemic. Specialists support would only be required in more complex OSA presentations analogous to current care for refractory hypertension or brittle diabetes. As discussed above this care pathway could be well supported through specialist collaboration via telemedicine [30,31,32]. 

Analysis of EMR data demonstrated only a 2.3% prevalence of OSA based on ICD-10 codes. Our previous work estimated a 25% OSA prevalence in the WV adult population while analysis of the WV Medicaid database (>400,000 patients) demonstrated that only 8.8% of the population had an OSA diagnosis based on claims data [15]. A 2.3% prevalence in the current study suggests a significantly larger OSA care gap in challenged rural WV communities primarily served by FQHCs than previously described. This finding further demonstrates the urgent need for a redesign of the current OSA care model. These communities likely have nuanced deficiencies that will require out of the box thinking to address. Unfortunately, evidence-based practice guidelines are not developed specifically for rural communities and few guidelines have been adapted to meet the needs of rural dwelling patients. As discussed by Afifi et al., robust community engaged approaches and implementation science will be required for health equity in these regions [33]. 

Despite the high disease burden of OSA worldwide and growing evidence of its negative health impacts, many healthcare systems have failed to adopt effective diagnostic and management strategies to address this growing problem. This care gap is amplified in rural WV communities (and likely other rural communities) and of critical importance given the high prevalence of comorbid cardiopulmonary conditions. The specialty workforce is very limited and cannot alone feasibly address the growing OSA problem. Engaging primary care providers in OSA diagnosis and management will be essential to addressing health disparities in WV and other highly vulnerable regions with limited healthcare resources. The current work provides insight into barriers and facilitators to OSA care from rural providers’ perspectives. Community engagement studies such as the current study will be essential to the creation of feasible, practical, relevant and culturally competent care pathways for providers serving rural communities with OSA and other respiratory disease to achieve health equity. 

While our study provides important insight into the thoughts, beliefs, and barriers to OSA care in rural WV communities, our findings are subject to certain limitations. First, it was conducted at 4 small clinics within two FQHCs serving the southern coalfields of WV. The unique care challenges in this region—poverty, high comorbid disease burden, limited transportation access, few specialists—could limit the generalizability of our findings. However, the limited number of specialists and challenges in terms of the social determinants of health are shared by rural communities throughout the U.S. [4]. Second, although the majority of providers at each clinic participate in the focus groups, it is possible that the participants’ responses may differ from those who did not participate. Third, while there is precedent for utilizing ICD-10 billing codes in our EMR analysis to assess disease prevalence, this approach relies on accurate coding by providers [14,15,34,35]. There is always some degree of error in coding, but given the large number of patient visits (>21,000) this is unlikely to dramatically alter our results. Lastly, another limitation is that the EMR analysis only captures people who actually present for care.

Finally, the identification of OSA is only the first step in improving outcomes. We did not evaluate whether the small number of individuals diagnosed with OSA were effectively treated. Previous studies suggest poor outcomes of OSA treatment in rural communities; however, further analysis is required to assess treatment outcomes in our population Appalachian population [13,14,36].

## 5. Conclusions

Although prior evidence suggests that primary care providers can effectively take ownership of OSA management, there are many potential challenges to implementation, particularly in rural WV communities with significant health disparities. This study suggests a lack of provider confidence in the ability to diagnose and treat OSA effectively; specific knowledge gaps related to OSA epidemiology, symptoms and treatment; overreliance and lack of access to specialty care for OSA management; and failure of new technologies to permeate rural communities (portable sleep testing, APAP). Education directed toward the identified knowledge gaps and training on new technologies would likely give rural primary care providers the confidence to take a more active role in OSA diagnosis and management, and the providers in our study were open to this intervention. An integrated model of care that incorporates primary care providers, specialists and effective use of modern technologies will be essential to address the identified OSA care disparities in rural WV and similar communities across the U.S. Community engagement studies such as the current study will be essential to the creation of feasible, practical, relevant and culturally competent care pathways for providers serving rural communities with OSA and other respiratory disease to achieve health equity. 

## Figures and Tables

**Table 1:** Focus Group Guide for Rural Practitioners.

**Provider Knowledge and Beliefs of Sleep Apnea**
Tell me what you know about sleep apnea. Do you regularly screen patients for sleep apnea. Why or why not? Are you comfortable managing obstructive sleep apnea? Are you comfortable reviewing sleep study results? If you decide to treat sleep apnea what therapies do you offer And why? Are you comfortable managing CPAP therapy for sleep apnea?
**Provider Percieved Barriers to Osa Management in Rural Commmunities**
If you have concerns of sleep apnea, what steps do you take to confirm the diagnosis? Are there barriers to successfully diagnose patients with sleep apnea. If so what are they? Is there anything that helps you successfully diagnose patients with sleep apnea? Do you feel your patients are receptive or would be receptive to discussions on sleep apnea? If you were to develop a special program to help improve screening and treatment of sleep apnea in your community: What would that program look like? What things would you include?

**Table 2:** Characteristics of Providers Participating in Focus Groups.

**AGE Mean (SD)**	
Mean	53.0 years (12.5) [Range 31 years–75 years]
**Gender, N (Percent)**	
Male	3 (21.4%)
Female	11 (78.6%)
**Race, N (Percent)**	
Caucasian	14 (100%)
**Practice Type, N (Percent)**	
Advanced Practice Provider	8 (57.1%)
Physician	6 (42.9%)
**Practice Tenure, Mean (SD) [Range]**	
Total Years in Practice	16.2 (15.0) [less than one month to 43 years]
Total Years in Practice at FQHC	8.7 (13.5) [less than one month to 43 years]
**Degree N (Percent)**	
MD	5 (35.7%)
DO	1 (7.1%)
NP	6 (42.8%)
PA	2 (14.3%)

**Table 3:** Participants Answering OSAKA Knowledge Items Correctly (*n* = 14).

Item Number	Number and Percent Answer Correctly	Item Number	Number and Percent Answer Correctly
Item 1	14 (100%)	Item 10	13 (92.69%)
Item 2	10 (71.4%)	Item 11	14 (100%)
Item 3	4 (28.6%)	Item 12	14 (100%)
Item 4	8 (57.1%)	Item 13	11 (78.6%)
Item 5	12 (85.7%)	Item 14	14 (100%)
Item 6	13 (92.6%)	Item 15	11 (78.6%)
Item 7	12 (85.7%)	Item 16	12 (85.7%)
Item 8	4 (28.6%)	Item 17	9 (64.3%)
Item 9	12 (85.6%)	Item 18	14 (100%)

**Table 4:** Categorization of Themes and Subthemes with Representative Quotes.

Theme	Subtheme	Representative Quotes
Barriers to OSA Care Delivery (N = 94)	OSA Care Access	“And lack of transportation.”
	Provider Knowledge/Beliefs OSA	“I, uh, learned a little bit more about sleep apnea in the past several years from continuing education class. But I think there’s a lot I don’t know.”
	Cost of OSA Care	“I noticed even before I started doing those physicals that some insurers prefer to just do the overnight sleep study without, uh, referral to a specialist, and so then I was left holding the results and was not real happy about that. So, so to me, the barrier, the biggest barrier is cost and coverage.”
Facilitators to OSA care Delivery (N = 33)	Specialty Referral Access	They [specialist and DME] pretty much take care of all that stuff. As long as they do all that, then I’m pretty comfortable with that.”
	Patient Characteristics	“I’ve never had any patient that closed the door on that discussion [regarding OSA] with me in my practice.”
Community Based Care needs to improve OSA management in targeted rural areas (N = 19)	Community Programming	“A lot of them [patients] are these big burly gentlemen that are very manly, and they don’t want to say that, ‘Yeah. Well, maybe something’s not right. I’m not tired’. But yet they can’t lift five pounds, because they are so tired. So, you have this, no disrespect, but this ‘man mentality’ to some of these guys.”
	Provider Preferences	“I mean, it’s my responsibility, but since you’re conducting this study, I would be interested in more education on it [OSA and CPAP]”.
	Educational Needs	“I probably would consider learning how to manage it [OSA] more myself and prescribe CPAP.”

**Table 5:** National Estimate and Statewide OSA Prevalence Data Compared to Rural WV FQHC OSA Prevalence Data.

Data Source	OSA Prevalence
Expected WV Adult Medicaid OSA Prevalence from National Data Sources [15]	25%
Observed WV Adult Medicaid OSA Prevalence from State Database Analysis [15]	8.8%
FQHC Specific OSA Prevalence from Local EMR Database Analysis	2.3%

## Data Availability

The data quantitative data presented in this study are available on request from the corresponding author. The data are not publicly available due to data agreement between the Federally Qualified Health Center and the West Virginia University School of Public Health.
